# Protein-coated nanostructured surfaces affect the adhesion of *Escherichia coli*

**DOI:** 10.1039/d2nr00976e

**Published:** 2022-04-28

**Authors:** Pawel Kallas, Håkon Valen, Mats Hulander, Nikolaj Gadegaard, John Stormonth-Darling, Padraic O'Reilly, Bernd Thiede, Martin Andersson, Håvard Jostein Haugen

**Affiliations:** Department of Biomaterials, Institute of Clinical Dentistry, University of Oslo 0455 Oslo Norway h.j.haugen@odont.uio.no; Nordic Institute of Dental Materials 0855 Oslo Norway; Department of Chemistry and Chemical Engineering, Chalmers University of Technology 412 58 Göteborg Sweden; James Watt School of Engineering, University of Glasgow G12 8QQ Glasgow UK; Molecular Vista San Jose CA 95119 USA; Department of Biosciences, The Faculty of Mathematics and Natural Sciences, University of Oslo 0371 Oslo Norway

## Abstract

Developing new implant surfaces with anti-adhesion bacterial properties used for medical devices remains a challenge. Here we describe a novel study investigating nanotopography influences on bacterial adhesion on surfaces with controlled interspatial nanopillar distances. The surfaces were coated with proteins (fibrinogen, collagen, serum and saliva) prior to *E. coli-WT* adhesion under flow conditions. PiFM provided chemical mapping and showed that proteins adsorbed both between and onto the nanopillars with a preference for areas between the nanopillars. *E. coli-WT* adhered least to protein-coated areas with low surface nanopillar coverage, most to surfaces coated with saliva, while human serum led to the lowest adhesion. Protein-coated nanostructured surfaces affected the adhesion of *E. coli-WT*.

## Abstract 2

Developing new implant surfaces with anti-adhesion bacterial properties used for medical devices remains a challenge.

## Graphical abstract

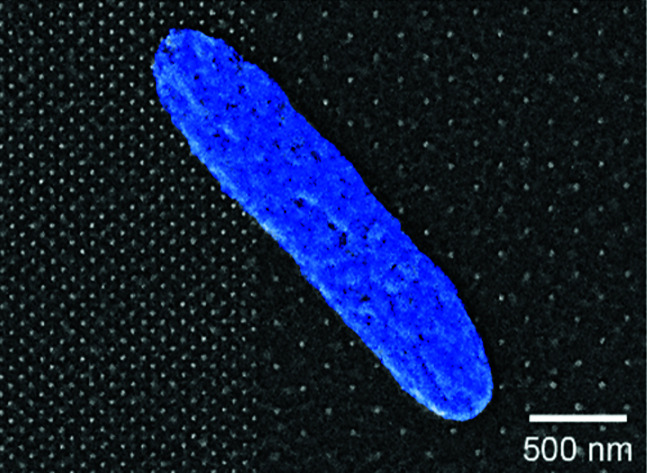

## Introduction

Adsorption of proteins from tissue fluids is the first event after implant placement. Proteins can be adsorbed from several bodily fluids, such as blood, plasma, or saliva if the biomaterial is placed orally.^1^ The adsorbed proteins mediate the adsorption of other molecules, the initial host cellular responses, host cell adhesion, and bacterial adhesion.^2–5^ Therefore, cells or bacteria that adhere to a biomaterial surface adhere to this protein layer rather than the pristine material.^6^ A protein's size, charge, hydrophobicity, and structure affect its adsorption onto a biomaterial surface. Smaller proteins move faster and serve as primary surface conditioners, and larger proteins tend to bind steadier due to their larger surface area.^7^ Moreover, the composition of the adsorbed layer and the conformation of the adsorbed proteins are key mediators of cell behaviour that control bioactivity and communication.^8^

Fibrinogen, a predominant plasma protein, plays an essential role in blood haemostasis and is among the first proteins to adsorb on the surface of a biomaterial when it is in contact with blood.^9,10^ Fibrinogen adsorption mediates platelet and monocyte/macrophage adhesion.^10–12^ Horbett *et al.* even identified fibrinogen adsorption onto biomaterial surfaces as the single most crucial event determining the biocompatibility of implants in soft tissue and blood.^10^ In contrast to serum, fibrinogen is also found in low concentrations in saliva.^13^ Human whole saliva has a diverse proteome, where approximately 27% of the proteins are shared with the plasma proteome.^1^ The adsorption of proteins to oral biomaterials placed in contact with both soft and hard tissues can mediate the adhesion of bacteria and hamper the biomaterial–host interaction.^14^ This initial bacterial adhesion has been shown to be a critical event in foreign body infection pathogenesis^15^ and may lead to a biomaterial-associated infection (BAI).

BAIs are the cause of many chronic and medical device-related infections. The specific bacterial species involved varies depending on the site of biomaterial placement and factors such as the local environment.^16^ The adhesion of bacteria is a multi-stage process,^17,18^ where reversible non-specific physical forces mediate the first step to the material surface. More specific and stable interactions occur when intimate contact with the surface is enabled, and colonisation of the surface may follow.^19^

Controlling or inhibiting bacterial surface adhesion is vital for preventing BAIs, and tissue integration is often essential to prevent infection and should occur prior to any bacterial colonisation to ensure medical device success.^20,21^ However, surface nanotopography may be an important parameter that has shown to mediate cell responses,^22^ as it affects both protein adsorption and conformation of the adsorbed proteins.^23^ In addition, surface nanotopography can also affect bacterial adhesion.^24,25^

When investigating surface nanotopography and its effect on biology, a common challenge is the need for a high throughput nanotopography manufacturing method with high reproducibility, high quality, and reasonable production time and costs.^26^ One of the production techniques that could aid in this challenge is the combination of electron beam lithography (EBL)^27^ and injection moulding.^28^ In the present work, these two techniques were combined to produce many identical yet still complex nanoscale surface patterns in polycarbonate with nanopillar interspace distances of 100, 250, and 500 nm. However, direct visualisation of these nanostructured materials in the single-nanometre-scale range is challenging because of the sensitivity bottlenecks in the current state-of-the-art visualisation technologies.^29^ Furthermore, three-dimensional distributions of photoinduced fields at or beyond the nanometre scale are essential to understand these interactions between nanostructures, proteins and bacterial adhesion, and thus we utilise here state-of-the-art nano-IR photo-induced force microscopy (PiFM) to detect the presence of fibrinogen, collagen, and proteins in human saliva.

The main aim of this study was to investigate the role of surfaces with an ordered nanotopography in protein adsorption and its subsequent effect on *E. coli-WT* bacterial adhesion. The hypothesis was that the presence of proteins affects *E. coli-WT* bacterial adhesion to nanostructured surfaces.

## Results

### Surface characterisation

**Fig. 1:**
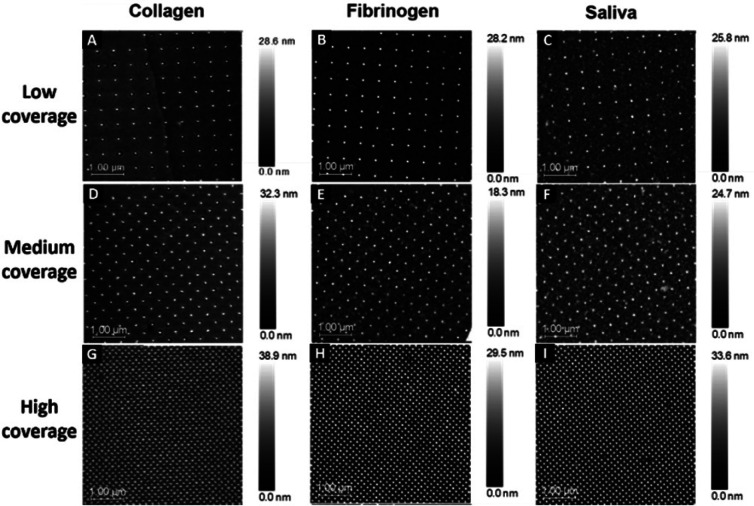
AFM images of protein-coated polycarbonate surfaces with different surface coverages: collagen (low coverage – A, medium coverage – D, and high coverage – G); fibrinogen (low coverage – B, medium coverage – E, and high coverage – H); saliva (low coverage – C, medium coverage – F, and high coverage – I).

This study used surfaces with injection moulded 40 nm diameter features in polycarbonate. Each pattern was divided into three sections with different surface coverages: low coverage (500 nm interspace distance), medium coverage (250 nm interspace distance), and high coverage of nanopillars (100 nm interspace distance). The distinct nanopatterns are visible through the thin protein layer on all surfaces: low (Fig. 1A–C), medium (Fig. 1D–F) and high coverage (Fig. 1G–I).

PiFM was used to confirm that the nanotopography was unchanged and similar to our previous measurement on uncoated nanotopographies, where pillar heights were found to be 25 ± 5 nm (described in ref. 30). Wettability analysis of uncoated surfaces showed an average contact angle of 67° ± 2°. Since the nanopatterned area of the surfaces was smaller than the size of the water droplet used to measure wettability, only the flat surface contact angle is presented.

**Fig. 2:**
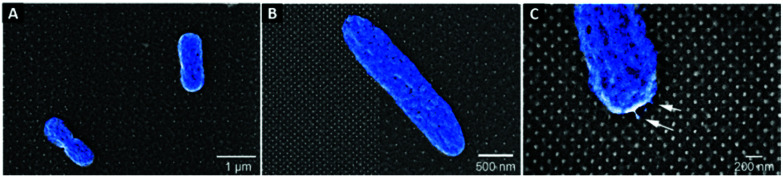
SEM images showing the attachment of *E-coli-WT* bacteria (falsely coloured blue) to the different topographies (A–C) with a layer of fibrinogen; noteworthy is the identified connections of the *E-coli-WT* to the nanopillars (white arrows (C)).

The protein layer filled the interpillar spaces. However, it was difficult to judge whether the nanopillars had proteins attached to them from the SEM characterisation. The nanopillars are visible at both nanometre and micrometre resolutions (Fig. 2A–C).

PiFM was utilised to detect the presence of fibrinogen, collagen, and proteins in human saliva, both at the interpillar space and on top of the nanopillars, as this technique also allows for chemical mapping, with nanoscale spatial resolution, acquired simultaneously with topography.

**Fig. 3:**
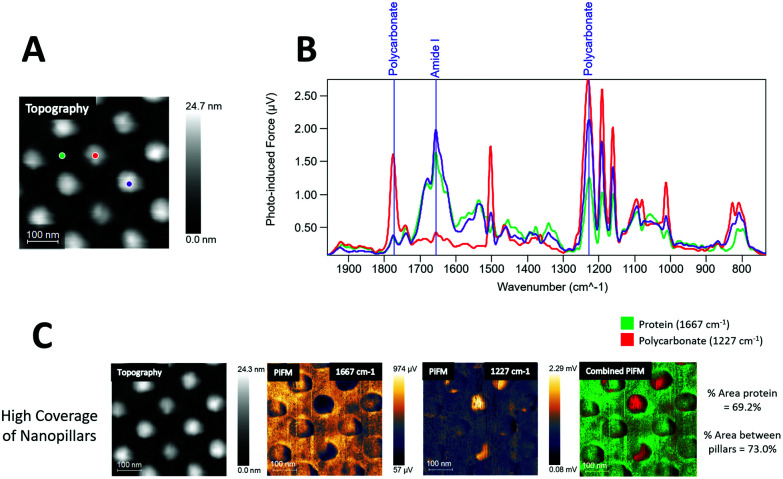
Topography and PiF-IR spectra from fibrinogen coated surfaces with high coverage of nanopillars. A – Topography signal channel with locations for IR sampling marked. B – Corresponding IR spectra from sample locations in A, where red and purple are collected from the top of nanopillars and green from the area in-between (see A). C – Large area scanning PiFM images of the fibrinogen coated surface with high coverage of nanopillars. Left is the topography channel, followed by amide I band (1667 cm^−1^) absorption, followed by detection of signature absorption from polycarbonate (1227 cm^−1^), and far-right the combined channels with percentage area protein coverage.

In Fig. 3A an image of the topography channel is shown together with labels for individual sampling sites for IR spectra on the fibrinogen coated samples. Data collected from the fibrinogen coated samples showed strong amide I absorption (1667 cm^−1^) from areas between the nanopillars and, to some extent, on top of the pillars (purple), as seen in the PiF-IR spectra in Fig. 3B. However, when viewed with large area scanning, fibrinogen seemed to preferentially adsorb on the flat regions between the nanopillars as nanopillars that showed strong absorption from polycarbonate (1227 and 1775 cm^−1^) was found and is also evident in Fig. 3C, middle and far right.

**Fig. 4:**
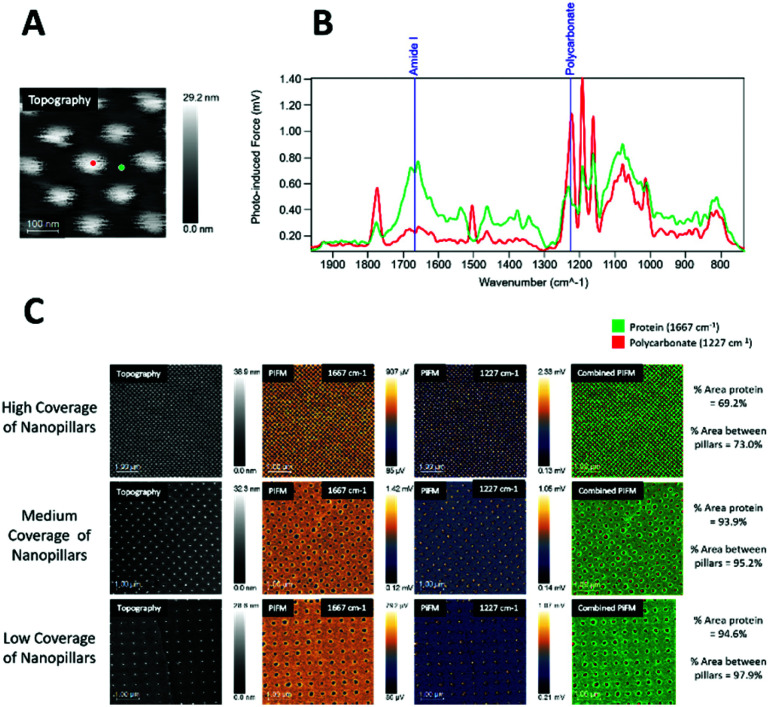
Topography and PiF-IR spectra from collagen-coated surfaces: A – Topography signal channel with locations for IR sampling marked. B – PiF-IR spectra for the tested sample, where red – nanopillar, and green – areas between nanopillars; C – Large area scanning PiFM images for three different surface coverages of nanopillars, with percentage area protein coverage of protein for each to the right.

On the collagen-coated samples (Fig. 4), both topography channel images, PiF-IR absorption spectra, and large area scanning images were collected for high, medium, and low coverages of nanopillars. Fig. 4B shows the spectra taken from two locations on the surfaces labelled with red and green dots, respectively, in Fig. 4A. Intense absorption in the amide I band (1667 cm^−1^) absorption was found in the areas between the nanopillars (green) and to a lesser degree on top of the pillars (red), where absorption from polycarbonate (1227 cm^−1^) dominated. The distinct peaks from the stretching vibrations of amide I of the C=O bond (1667 cm^−1^) and in-plane N–H bending amine II (1505 and 1580 cm^−1^)^31^ did not interfere with the signature peaks in polycarbonate (carbonyl stretching vibration peak at 1775 cm^−1^), and the O–C–O stretching mode triplet at 1152, 1188 and 1227 cm^−1^.^32,33^ The signature peaks of polycarbonate were characterised by the carbonyl stretching vibration, which produced a robust and well-resolved peak at 1775 cm^−1^, while the O–C–O stretching mode caused a very intense triplet at 1152, 1188 and 1227 cm^−1^.^32,33^ 1227 cm^−1^ was chosen to represent polycarbonate as it had no interference with the protein spectra. Either of these wavenumbers could be used to verify the presence of protein on the surface and nanopillar (Fig. 4A and B), as amide I is the most intense absorption band in proteins primarily governed by the stretching vibrations of the C=O. Amide II is typically found for wavenumber regions 1505 and 1580 cm^−1^, mainly from in-plane N–H bending.^31^

The PiFM chemical images in Fig. 4C show the intensities of 1667 cm^−1^ (amide I) and 1227 cm^−1^ (polycarbonate) and in the last row the merging of these two images for high, medium and low coverage nanostructures. The protein layer was evenly distributed on all surfaces, and the top of the pillars had generally less protein than the area between the pillars.

The area percentages of protein surface coverage for collagen were found to be directly proportional to the flat regions between the nanopillars. The highest protein coverage was observed for the low coverage of nanopillars (94.6%), while the lowest protein coverage was observed for the high coverage of nanopillars (69.2%).

**Fig. 5:**
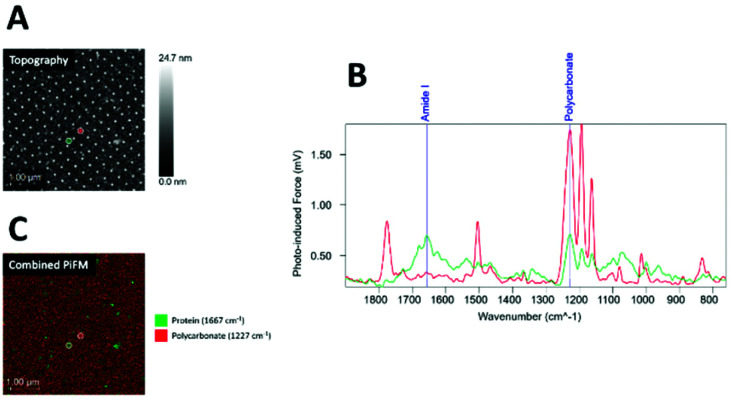
Topography and PiF-IR spectra of saliva on tested surfaces. A – Topography image of a surface with high coverage of nanoparticles where red – nanopillars, green – areas between nanopillars; B – IR absorption spectra from the highlighted sample locations in (A), where red – nanopillars, green – areas between nanopillars; C – combined PiFM image for amide I and polycarbonate signature absorption.

Samples coated with human saliva (Fig. 5) showed a less dominant IR absorption peak in the amide I region (1667 cm^−1^) compared to those coated with fibrinogen and collagen. Although absorption in the amide I region was detected both on and between the nanopillars, some absorption from polycarbonate at 1227 cm^−1^ was detected throughout the surface, indicating a thinner protein layer.

**Fig. 6:**
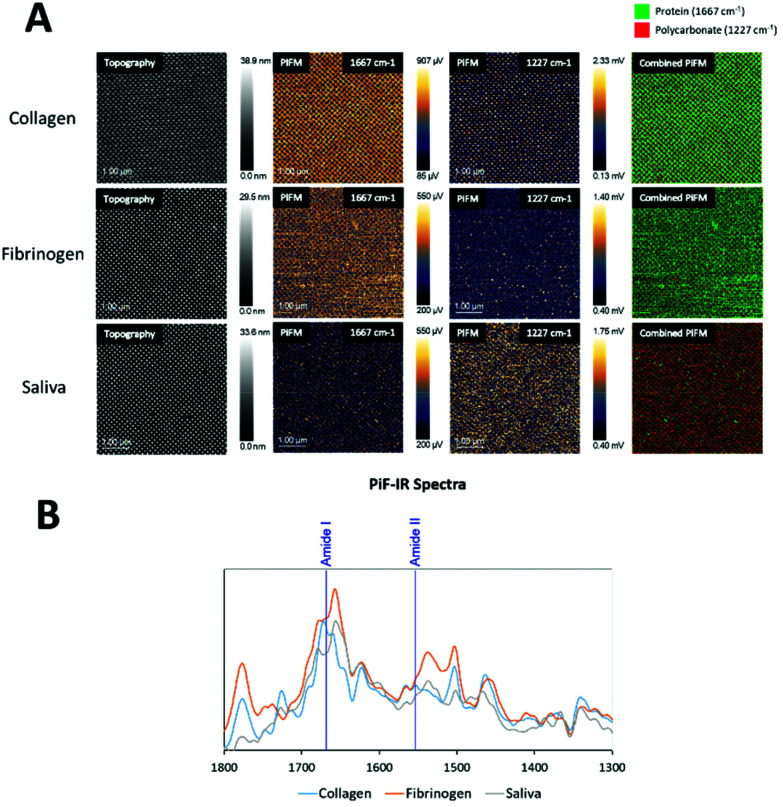
Combined PiFM (A) and PiF-IR spectra (B) comparison for collagen, fibrinogen, and saliva.

The fibrinogen sample showed the highest protein coverage, followed by collagen, whereas saliva showed less coverage (Fig. 6A).

### Proteome analyses of protein adsorption using LC-MS

LC-MS was performed to identify which proteins from human serum and saliva were adsorbed onto the sample surface prior to the bacterial adhesion study. For label-free quantification of the LC-MS data, three surfaces separately exposed to human serum and saliva were compared to an unexposed control surface. In addition, solutions of serum and saliva were used as positive controls.

171 protein groups were identified in saliva and 149 in serum. The ten most significant adsorbed proteins onto the nanostructures for saliva and serum, respectively, are summarised in Tables 1 and 2. The list is ordered by the most significantly identified proteins with the highest scores (−10 lg *P*) detected by LC-MS. In addition, the amino acid sequence coverage of the detected peptides and the number of peptides are displayed. Glyceraldehyde-3-phosphate dehydrogenase and cytoplasmic actin were the two most adsorbed proteins from saliva (Table 1). Apolipoprotein B-100 and complement C3 were the two most adsorbed proteins from serum (Table 2).

**Table 1:** Ten most significantly identified proteins with the highest scores (−10 lg *P*) in human saliva proteins

No.	Accession	Name	−10 lg *P*	Sequence coverage (%)	#Peptides
1	P04406	Glyceraldehyde-3-phosphate dehydrogenase	342	54	20
2	P60709	Actin, cytoplasmic	317	49	18
3	P02788	Lactotransferrin	317	23	22
4	P14618	Pyruvate kinase	305	40	21
5	P35908	Keratin, type II cytoskeletal 2 epidermal	229	18	10
6	P04264	Keratin, type II cytoskeletal 1	229	22	12
7	P04075	Fructose-bisphosphate aldolase A	228	29	9
8	Q9UBC9	Small proline-rich protein	225	47	9
9	P52209	6-Phosphogluconate dehydrogenase, decarboxylating	216	15	8
10	P01876	Immunoglobulin heavy constant alpha 1	213	20	7

**Table 2:** Ten most significantly identified proteins with the highest scores (−10 lg *P*) in human serum proteins

No.	Accession	Name	−10 lg *P*	Sequence coverage (%)	#Peptides
1	P04114	Apolipoprotein B-100	565	38	151
2	P01024	Complement C3	470	35	48
3	P06727	Apolipoprotein A-IV	428	66	41
4	P02647	Apolipoprotein A-I	413	74	33
5	P06396	Gelsolin	402	41	23
6	P19823	Inter-alpha-trypsin inhibitor heavy chain H2	381	38	26
7	P02649	Apolipoprotein E	373	74	27
8	P01023	Alpha-2-macroglobulin	361	26	29
9	P01009	Alpha-1-antitrypsin	351	44	18
10	Q92954	Proteoglycan 4	333	13	19

The list is ordered by the most significantly identified proteins with the highest scores (−10 lg *P*) detected by LC-MS onto the nanostructured surfaces in terms of (−10 lg *P*). In addition, the amino acid sequence coverage of the detected peptides and the number of peptides are displayed.

**Fig. 7:**
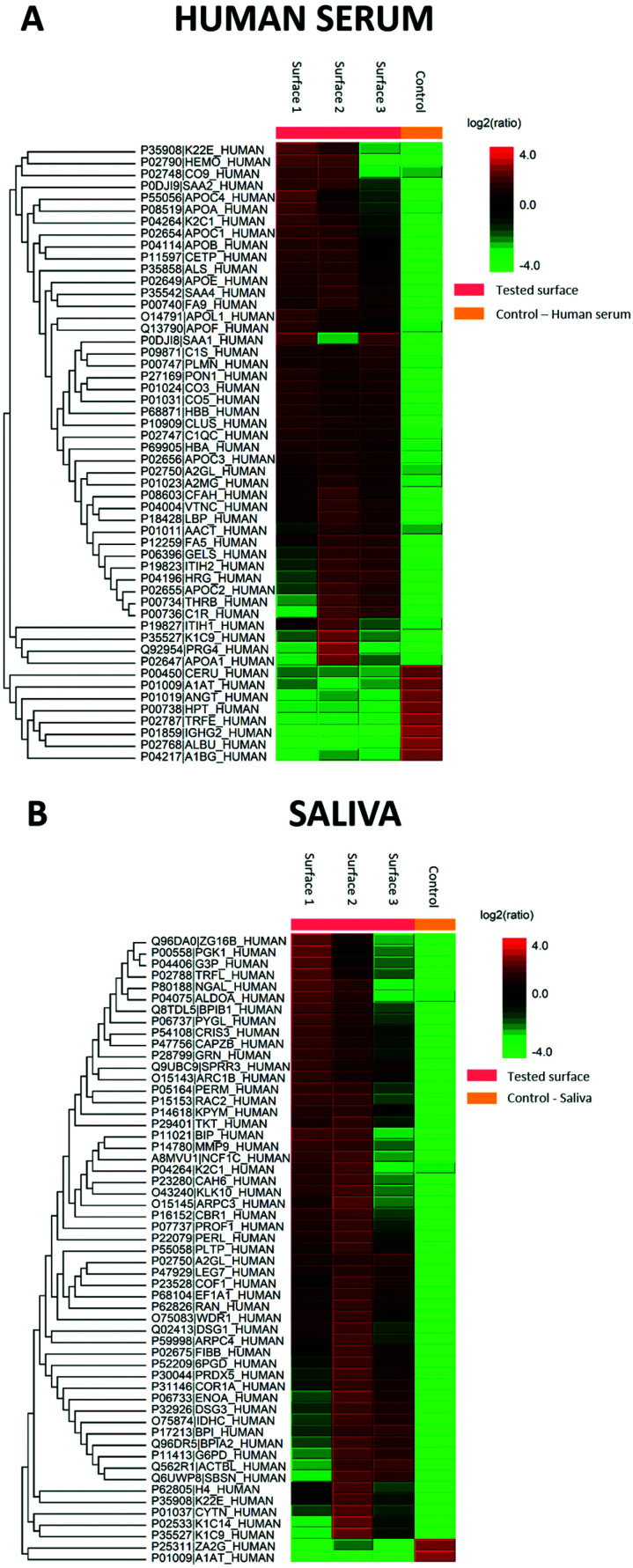
Quantitative proteome analysis of proteins present on human serum and saliva surfaces: A – heat map representation of quantified human serum proteins (>two-fold change, 1% FDR); B – heat map representation of quantified saliva proteins (>two-fold change, 1% FDR). As shown in the colour scale bar: red shades – increased protein expression; green shades – reduced protein expression; tested surfaces are from the protein-coated nanopatterned surfaces and control from the neat human serum/saliva.

The generated heat maps (Fig. 7A and C) present an overview of the relative protein abundance adsorbed on the surfaces compared to control human serum and saliva. The red shades indicate high protein abundance, while green shades indicate low protein abundance. The heat maps show the proteins to be different in intensity by more than two-fold and a permutation FDR (false discovery rate) of 1% (56 protein groups in both). Excluding keratins, which are common contaminants in proteomics experiments, two proteins were found to be changed in both sample types. The average molecular mass of the proteins in saliva was 50 385 Da for all 171 protein groups, 47 809 Da for the 48 proteins increased on the surface, and 46 986 Da for the eight decreased proteins. In contrast, the average molecular masses were more diverse in serum with 67 253 Da for all 149 protein groups, 46 986 for the nine decreased proteins, and 84 684 Da for the 47 surface increased proteins.

**Fig. 8:**
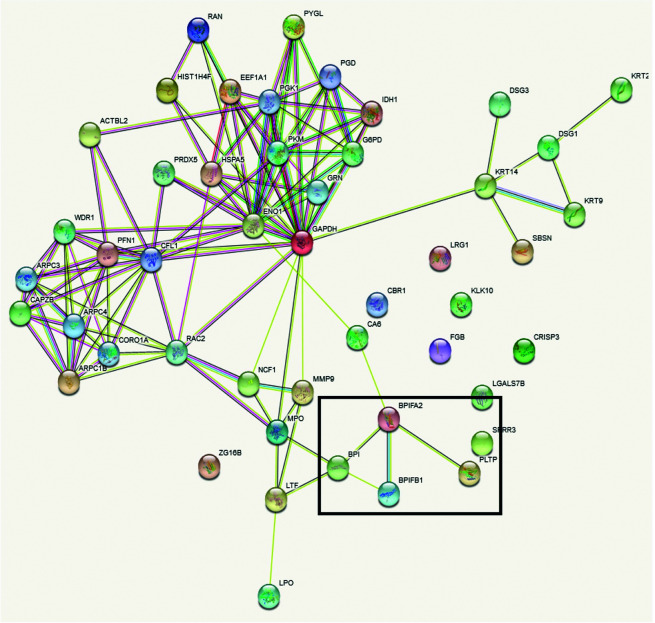
Protein–protein interaction analysis of upregulated proteins in saliva using STRING.

Functional annotation cluster analysis of the upregulated proteins using DAVID Bioinformatic Resources 6.8 with high stringency revealed the complement pathway (enrichment score 6.38, nine proteins), chylomicron/cholesterol metabolism (enrichment score 6.37, six proteins), and blood coagulation (enrichment score 5.27, five proteins) were found for the 47 upregulated proteins in serum. In contrast, cell–cell adhesion (enrichment score: 3.78, seven proteins), lipid-binding proteins (enrichment score 3.75, four proteins) and metabolic pathways (enrichment score 3.27, nine proteins) were obtained as the most significant term for the 51 proteins in saliva. In comparison to the DAVID analysis of all identified saliva proteins, the four lipid-binding proteins (BPI fold containing family A member 2 (BPIFA2), BPI fold containing family B member 1 (BPIFB1), bactericidal permeability-increasing protein (BPI), and phospholipid transfer protein (PLTP)) seem to be specifically enriched. In addition, the protein–protein interaction map of the upregulated saliva proteins analysed using STRING 11.5 also revealed that these four proteins are connected (Fig. 8).

### Bacterial adhesion

We observed similar patterns between nanostructured surfaces with increased *E. coli-WT* adhesion to areas with a higher surface coverage of nanopattern regardless of which protein was present on the surface before the bacterial adhesion.

**Fig. 9:**
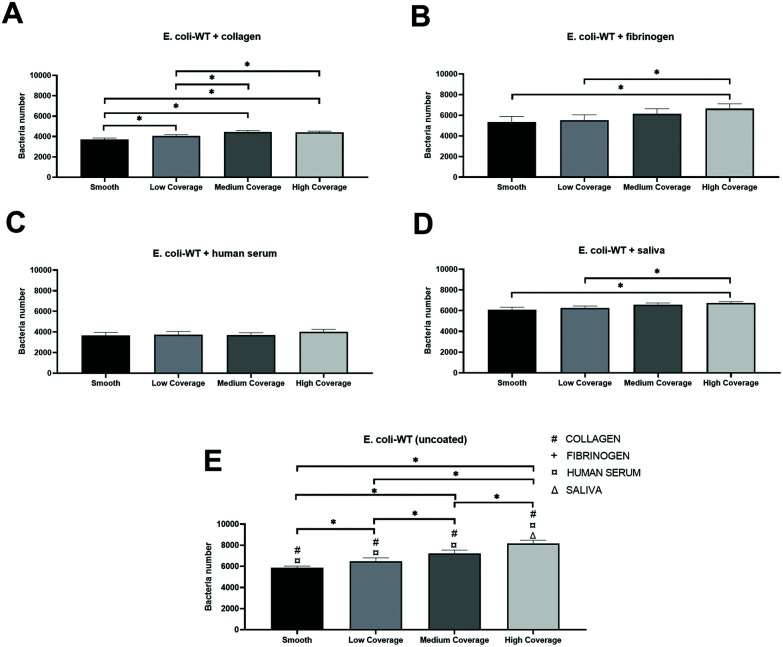
*E. coli-WT* adhesion to surfaces coated with A – collagen; B – fibrinogen; C – human serum; D – saliva; and E – uncoated surfaces with significance (*p*-value <0.05) between equivalent coated surfaces with nanopillars: # – collagen, +– fibrinogen, ¤ – human serum, and Δ – saliva. Significant at * *p*-value <0.05, with the standard error of the mean, *n* = 56. Bacteria number was calculated per 0.71 mm^2^ for each surface coverage of nanopatterns on all replicates (*n* = 56).

The highest total adhesion was observed for surfaces coated with saliva (Fig. 9D). The number of adhered bacteria gradually decreased by 3% on medium coverage, 7% on low coverage, and 10% on the smooth control part of the surface compared to high coverage of nanopatterns. However, there was a significant difference between the smooth and high coverage and low and high coverage.

The lowest total adhesion was observed for surfaces coated with human serum (Fig. 9C), where 4015 cells adhered to the high coverage part of the surface. We also observed a slightly lower adhesion to medium coverage (8% decrease from high coverage) compared to low coverage (7% decrease from high coverage), with the lowest adhesion to the smooth part of the surface (9% decrease from high coverage). There was no significant difference between the tested groups.

We observed the most significant differences between the numbers of adhered bacteria on surfaces coated with collagen (Fig. 9A). Fibrinogen-coated surfaces (Fig. 9B) showed the same significant differences observed in saliva samples and the highest decrease in adhesion – by 19% in the smooth area compared to high coverage.

## Discussion

After implantation, the initial host response to biomaterial surfaces is the adsorption of proteins from blood and interstitial fluids. This adsorbed protein layer modulates the bacterial attachment to biomaterials and is pivotal for the long-term success of an implant.^34^ Implant surfaces are typically covered with collagen, fibrinogen, human serum, or saliva immediately upon implantation if placed in the oral cavity.^35^ Therefore, these proteins were pre-coated to our experimental surfaces to study their effect on bacterial adhesion to our nanopatterned surfaces. In addition, topographical features at nanoscale levels may significantly affect the extent of bacterial adhesion.^36^ This study investigated the adhesion of wild-type *Escherichia coli* on different protein-coated surfaces that featured three different nanotopographies. The design of the nanopatterns was inspired by naturally occurring anti-bacterial surfaces that often consist of nanopillars of diameter ∼50–250 nm, with different heights and densities.^37^ Recent observations show that such natural nanostructured surfaces may kill bacteria by rupturing the cell wall, known as the contact killing mechanism.^38^

Our previous work described that electron beam lithography combined with injection moulding could be used to design nanostructured polymeric surfaces at low cost and high speed. It also helped us create a high number of reproducible, identical surface topographies on a reduced number of substrates.^28^

PiFM is one of the few techniques that combine IR spectroscopy with AFM to obtain both non-destructive topographic and chemical information with sub ∼10 nm resolution simultaneously *via* tip-enhanced near-field imaging and spectroscopy through bi-modal AFM.^39,40^ PiFM nano-scale chemical mapping is one of the most advanced mapping technologies that creates chemical absorption maps of nano-scale features.^41^ For organic samples, such as those used in this study, the photo-induced force is dominated by a thermally expanded sample's modulated van der Waals force gradient due to IR absorption; the more significant the absorption at a given wavenumber, the greater the attractive photo-induced force.^42^ PiFM is, apart from s-SNOM,^43^ the only technique to quantify and detect the adherence of proteins to specific nanostructures chemically. Here we could verify that PiFM provided protein mapping in areas both between and on top of the nanopillars as the mapping displayed a strong amide I peak (stretching vibrations of the C=O: 1667 cm^−1^) and evidence of the amide II peak (in-plane N–H bending: 1505 cm^−1^), typically used to identify proteins.^31^ This enabled us to compare the adsorption of various f proteins on the nanopillars, which are only a few nm in diameter. More fibrinogen was found on the top of the pillars than collagen, indicating stronger interactions between fibrinogen and nanostructures, as also documented by others.^23,44^ Finally, the proposed PiFM technique can beneficially apply further studies on nanotechnology, proteins, and bacterial interactions.

We used LC-MS/MS proteomics to identify and quantify proteins adsorbed from saliva and human serum on the nanostructured surfaces. We found negligible differences from sample to sample upon proteomics analysis, which ensured sample homogeneity. The adsorbed proteins from serum were comparable to previous studies.^45^ While this method does not allow examining protein adsorption at the nanoscale, it provides an overall view of the proteins adsorbed to the surface. The sequence coverages may not be a perfect indicator of the number of adsorbed proteins since small proteins more easily can yield a higher sequence coverage than large proteins.^46^ However, this could still be an indicator, as large proteins would take up a larger surface area upon adsorption.

The primary protein structure is related to the sequence of amino acids and has a substantial variation between proteins. Larger proteins have more binding sites to interact with the material surface and therefore have higher potential for adsorption on the surface.^47^ This study found increased average molecular masses of the proteins with more than a two-fold increase than all identified proteins in serum but not in saliva. The mass transfer rate of protein molecules in complex protein solutions to the surface is directly related to their concentration and inversely to their molecular weight. This applies to solutions that contain hundreds of different proteins, such as human serum and saliva used in our study.^48^ Lehnfeld *et al.*^49^ showed that maximum protein adsorption on chemically modified silica surfaces occurred after one hour for saliva and 10 min for the much higher concentrated human serum. However, they observed that adsorbed proteins did not correlate linearly with surface physicochemical parameters. Protein adsorption is divided into the following steps: transport, adsorption, and desorption, which were in the transport stage, distribution being the dominating step.^50,51^ For a multiprotein system, small proteins distribute faster and arrive at the surface earlier than the large ones, and therefore are repelled by larger molecules. Although this displacement phenomenon, known as the Vroman effect,^52^ has been researched extensively, this effect still cannot explain our findings here. As the protein size and structure are important factors affecting the adsorption process, the stronger binding of the protein to the surface applies to larger proteins.^53^ The protein's charge distribution is likely to affect the surface affinity. It has been experimentally shown that proteins exhibit more significant adsorption at or near their isoelectric pH because of the minimised charge repulsion factor among the adsorbed molecules. Both size, net charge, and structure of a protein are other significant parameters to control its adsorption.^54^ In type 1 collagen, the fundamental structural unit is 280–300 nm long and 1.5 nm in diameter,^55^ while the fibrinogen molecule size is 47.5 ± 2.5 nm long and 0.5–0.7 nm in diameter.^56^ In this regard, collagen should have had higher affinity to the surface than fibrinogen, although our results showed the opposite. However, Denis *et al.*^57^ investigated collagen adsorption on substrates with controlled topography and surface chemistry. They observed that nanoscale protrusions inhibited collagen mobility, while the molecules were relatively free to move and assemble on smooth surfaces.

We choose *E. coli* wild type as bacterial species in this study due to its known affinity to surgical site infections.^58,59^ We observed that the different adsorbed proteins affected bacterial adhesion. The highest adhesion of *E. coli-WT* was observed on surfaces coated with saliva. These surfaces were the least coated with proteins, as a stronger signal from polycarbonate was found on the saliva-coated surfaces in our PiFM measurements. The protein signals from saliva samples were comparable to collagen-coated surfaces (third-most *E. coli-WT* adhered surfaces), whereas the surfaces coated with fibrinogen were the second most *E. coli-WT* adhered surfaces in our study. Both fibrinogen and collagen samples showed a strong protein signal for areas between the nanopillars. An additional explanation for differences in bacterial adhesion observed on tested surfaces may lie in the isoelectric point of the tested proteins. For example, unmodified *E. coli* cells show a surface isoelectric point of pI = 5.6,^60^ which is similar to the isoelectric point of fibrinogen (pI = 5.8).^61^ These values are lower than the isoelectric point of collagen, pI = 7.2.^62^ Therefore, we observed higher bacterial adhesion on surfaces coated with fibrinogen rather than collagen due to the minimised charge repulsion factor.

Liu *et al.*^63^ suggested that increasing nanoscale roughness and surface hydrophilicity could increase the total protein adsorption. Several studies confirm a link between surface wettability and anti-biofouling effects,^64,65^ assigning the microbes’ non-stickiness to the hydrophobic nature. However, from the PiFM analyses, it was evident that fewer proteins adsorbed on top of the nanopillars for all protein solutions than on the surface in-between, regardless of the surface coverage of the nanopillars (Fig. 6). Additionally, *E. coli-WT* is a fimbriated bacterium that does not have to rely on its adhesion merely on cell surface proteins,^66,67^ and also does not seem to have any known integrins for epitopes on fibrinogen or collagen, which could explain the higher number of cells found on the surfaces pre-conditioned with saliva, where a low amount of proteins were detected. We observed a similar adhesion pattern in our previous study where the highest adhesion of *E. coli-WT* was seen for the areas with high coverage of nanopillars, even without protein coatings.^68^ However, we also observed a significant difference between the smooth and low coverage areas for uncoated surfaces (controls), which we had not observed in our previous study. This can be explained by the higher tested area of smooth surfaces without nanopillars in the current study and, therefore differences in flow between the corresponding surface coverages in these two studies. This indicates that on our nanopatterned surfaces, *E. coli-WT* adhesion only occurs on top of the nanopillars and is dependent on the number of available nanosized (protein-free) attachment points that the pillars provide.

## Experimental

### Preparation of nanostructured surfaces

Nanostructured polymer surfaces were prepared using electron beam lithography and injection moulding as previously described^68^ and are listed in Table 3. In short, a master template with nanopatterns was made by electron beam lithography followed by a replication process using UV-based nanoimprint lithography (UV-NIL) (EV Group, Sankt Florian am Inn, Austria) into a working stamp material.^69^ Injection moulding was performed using an Engel Victory 28 hydraulic injection moulding machine (Engel Austria GmbH, Schwertberg, Austria) to produce multiple polystyrene samples.^30^ Each surface consisted of 7 × 4 repetitions of the pattern divided into three sections (total size 1 × 3 mm) with a different surface coverage: low (2.5%), medium (3.5%) and high (20%). A total of 56 nanostructured surfaces with different coverages measured were made.

**Table 3:** The characterisation of the nanopillars with interspace, diameter, and height (*n* = 5)

Nomenclature	Pillar coverage (%)	Pillar interspacing (nm)	Pillar diameter (nm)	Pillar height (nm)
Low	2.5	500	40	25 ± 5
Medium	3.5	250	40	25 ± 5
High	20	100	40	25 ± 5

### Protein preparation and surface coating

Four types of protein solutions were used for the surface coating to investigate the role of protein in bacterial adhesion. Saliva was collected during masticatory stimulation with paraffin pellets (Ivoclar Vivadent AG, Schaan, Liechtenstein) from five healthy donors who were abstained from eating and drinking for 1 h prior to the collection. 25 mL of saliva was cleared in a polypropylene tube by centrifugation at 5000 rpm for 5 min at 21 °C. The supernatant was filtered through a 0.22 μm membrane filter (TPP Techno Plastic Products AG, Trasadingen, Switzerland). The sterile saliva samples were stored in a freezer at −70 °C and defrosted before the coating. Human serum was obtained from Sigma-Aldrich, Oslo, Norway, and the aliquots were stored at −20 °C until use for adsorption experiments. 100 μg mL^−1^ human fibrinogen (Sigma-Aldrich, Oslo, Norway) in phosphate-buffered saline (PBS, Lonza, Verviers, Belgium), and 100 μg mL^−1^ collagen type-1 solution (Advanced BioMatrix, Inc., Carlsbad, CA, USA) in PBS were separately prepared prior to the surface coating. Under aseptic conditions, each nanopatterned surface was covered with 1 mL of each protein solution and incubated for 60 min at room temperature, washed with PBS and then used immediately for bacterial adhesion experiments.

### Surface characterisation

Water contact angle measurements were performed on the experimental surfaces using a 100-00-230 NRL contact angle goniometer (Ramé-Hart Inc. Mountain Lakes, NJ, USA). A 5 μL Milli-Q water droplet was applied to the surface. The average contact angle was measured based on seven measurements at 30 s time points.

Nano-IR photo-induced force microscopy (PiFM) was conducted to detect the presence of fibrinogen, collagen, and proteins in human saliva using a VistaScope microscope from Molecular Vista Inc. (San Jose, CA, USA), coupled with a LaserTune quantum cascade laser (QCL) from Block Engineering, with a range of 733 to 1960 cm^−1^ and a spectral linewidth of 2 cm^−1^. The laser beam was focused on the interface between the sample and a metallic atomic force microscopy (AFM) tip, operated in dynamic non-contact mode, *via* a parabolic mirror. The laser was modulated at a frequency, *f*_m_, so that *f*_m_ = *f*_1_ − *f*_0_, where *f*_0_ and *f*_1_ are the 1^st^ and 2^nd^ mechanical resonance modes of the cantilever, and this is referred to as the sideband mode and takes advantage of the quality factor of the cantilever to increase the sensitivity.^40^ During imaging, the average laser power on the sample surface was 100 μW, with an elliptical spot size from the off-axis parabolic mirror of approximately *λ* × 1.5*λ*. For all measurements, platinum/iridium-coated NCH 300 kHz non-contact cantilevers from Nanosensors were also used. The image and data processing for the PiFM and topography images were performed using SurfaceWorks. All PiFM and topography images were acquired with 256 × 256 pixel resolution and a scan speed of 0.39 line per s. Each point spectrum had a spectral acquisition time of 45 s per spectrum (3 × 15 s averaged) over a range of 760 to 1900 cm^−1^ and is power normalised. All imaging was performed by Molecular Vista Inc. (San Jose, CA, USA).

### Proteomics protein adsorption characterisation

Liquid chromatography-mass spectrometry (LC-MS/MS) was performed to analyse human serum and saliva protein content. The adsorbed proteins on each nanostructured surface were dissolved in 10 μl of 0.1% formic acid/2% acetonitrile. There were four samples in 2 groups evaluated – 3 samples of surfaces covered with proteins and one control sample (without nanostructures). 5 μl was analysed using an Ultimate 3000 RSLCnano-UHPLC system connected to a Q Exactive mass spectrometer (Thermo Fisher Scientific, Bremen, Germany) equipped with a nano-electrospray ion source. For liquid chromatography separation, an Acclaim PepMap 100 column (C18, 2 μm beads, 100 Å, 75 μm inner diameter, 50 cm length) (Dionex, Sunnyvale CA, USA) was used. A flow rate of 300 nL min^−1^ was employed with a solvent gradient of 4–35% B in 60 min. Solvent A was 0.1% formic acid and solvent B was 0.1% formic acid/90% acetonitrile. The mass spectrometer was automatically operated in the data-dependent mode to automatically switch between MS and MS/MS acquisition. Survey full-scan MS spectra (from *m*/*z* 400 to 2000) were acquired with the resolution *R* = 70 000 at *m*/*z* 200, after accumulation to a target of 1e6. The maximum allowed ion accumulation times were 60 ms. The method used allowed the sequential isolation of up to ten most intense ions, depending on the signal intensity (intensity threshold 1.7e4), for fragmentation using higher-energy collision induced dissociation (HCD) at a target value of 1e5 charges, an NCE of 28, and a resolution *R* = 17 500. Target ions already selected for MS/MS were dynamically excluded for 30 s. The isolation window was *m*/*z* = 2 without offset. The lock mass option was enabled in MS mode for accurate mass measurements. Proteins were identified using the database UniProt_SwissProt with Homo sapiens taxon in PEAKS viewer Xpro version (Bioinformatics Solutions Inc., Waterloo, Canada). Heatmaps were made in the same program. No coefficient of variation filter was used, and no outliers were removed.

### Bacterial adhesion

*E. coli BW25113 7636* (later referred to as *E. coli-WT*) were grown in tryptic soy broth (TSB, Sigma-Aldrich, Oslo, Norway) medium overnight at 37 °C in centrifuge tubes and under a 5% CO_2_ atmosphere. The overnight culture was diluted 10 times in the morning and left to grow again under the same conditions until the optical density reached OD_600_ = 1 (Thermo Scientific Spectronic 200E, Waltham, MA, USA). After that, the samples were centrifuged at 5000 rpm for 5 min at room temperature to obtain a pellet. The supernatant was discarded and exchanged for PBS to obtain OD_600_ = 1 and the suspension was then used immediately for the adhesion experiments.

As previously described, the bacterial adhesion experiment was performed in a laminar flow chamber.^68^ After placing the protein-coated sample in the flow chamber, the system was flushed with distilled water for about 1 min at a constant 20 mL min^−1^ flow to remove any air bubbles trapped in the system. 10 mL of the bacterial solution was then manually injected into the system using a syringe. The valves were then closed, and the bacteria were let to adhere under static conditions for 5 min at room temperature. This procedure was followed by manually injecting 10 mL of 0.01% acridine orange (AO) to stain the cells for later viewing with fluorescence microscopy. After 3 min of staining, the valves were open again, and the sample was flushed for 5 min with distilled water at the same flow rate as before (20 mL min^−1^). The measurements were repeated two times on two occasions on 56 samples in total.

The flow chamber was transferred to a fluorescence light microscope (Olympus BX51, Olympus Optical, Tokyo, Japan). Images were obtained at a magnification of 90× by using a 10× magnification objective with a U-MNB2 filter (excitation BP 470–490 nm and emission LP 520 nm), and one image was obtained for each surface coverage of nanopatterns on all replicates (*n* = 56). Image analysis was performed using ImageJ software version 1.53a (NIH, Bethesda, MD, USA). The bacterial coverage of the nanopatterns was calculated using an ImageJ plugin^70^ as previously described,^68^ and the bacterial number was calculated per 0.71 mm^2^ for each surface coverage of nanopatterns on all replicates (*n* = 56).

### Statistical analysis

Statistical analysis was performed using GraphPad Prism version 6.07 (GraphPad Software, La Jolla, CA, USA). All datasets were tested for normality before analysis. The effect of the different protein-coated surface coverages for *E. coli-WT* adhesion was analysed using one-way ANOVA with repeated measurements, followed by Tukey's multiple comparison test. To compare the uncoated surfaces (controls) with protein-coated ones, a two-way ANOVA with repeated measurements was performed followed by Tukey's multiple comparisons test for the simple effects within each column, and the number of adhered *E. coli-WT* was compared to each other for each surface coverage. Data were presented as mean with the standard error of the mean. The results were considered significant with a *p*-value ≤0.05. The total number of analysed surface coverages was *n* = 56.

## Conclusions

In this study, a systematic evaluation was performed where nanostructured pillars with controlled interspatial distances were used to examine their role in the quantity and type of protein being adsorbed from four different protein solutions. In addition, we successfully visualised a 3D chemical mapping with nano-IR photo-induced force microscopy that detected the presence of fibrinogen, collagen, and proteins in human saliva on and between nanopillars. Finally, the technique showed that the protein adsorption was higher between the nanopillars than on top.

The protein-coated nanostructured surfaces were subjected to *E. coli-WT* to examine the relationship between nanotopography, protein adsorption and bacterial adhesion. In general, the presence of proteins decreased the adhesion of *E. coli-WT* to nanopatterned surfaces, and an increase in interpillar distance was associated with reduced bacterial adhesion. Hence, we found a correlation between nanostructures and bacterial adhesion. The results from the study provide insight into the development of new implant surfaces with anti-adhesion bacterial properties used for medical devices.

Based on our observations, we envision that the ideal principle of surface design for developing new implant surfaces with bacterial anti-adhesion properties should rely on the surface with the least nanofeatures, such as nanopillars.

## Author contributions

Conceptualization: H. J. H., H. V., and N. G. methodology: M. H., H. J. H, H. V., and N. G.; formal analysis, data curation, and investigation: P. K., J. S. D., P. O., and B. T.; writing – original draft preparation: P. K. and H. J. H.; writing – review and editing: M. H., H. J. H., P. O., and H. V.; supervision: M. A., M. H., and H. V.; and project administration: H. J. H. and H. V. All authors have read and agreed to the published version of the manuscript.

## Conflicts of interest

There are no conflicts to declare, except for Padraic O'Reilly who is employed at Molecular Vista Inc., which produces one of the equipment (PiFM) used in this study.

## Supplementary Material
